# Resident CD24 ^+^LCN2 ^+^ LPCs aggravate fibrosis and inflammatory progression via the recruitment of TPPP3 ^+^COL10A1 ^+^ macrophages in NASH: CD24 ^+^LCN2 ^+^ LPCs aggravate fibrosis and inflammatory progression

**DOI:** 10.3724/abbs.2025081

**Published:** 2025-05-16

**Authors:** Min Ding, Xiaoshu Qi, Weijian Huang, Yan Lin, Hexin Yan

**Affiliations:** 1 Department of Interventional Oncology Renji Hospital Shanghai Jiaotong University School of Medicine Shanghai 200127 China; 2 Department of Anesthesiology and Critical Care Medicine Renji Hospital Shanghai Jiaotong University School of Medicine Shanghai 200127 China; 3 Shanghai Celliver Biotechnology Co. Ltd. Shanghai 200120 China; 4 Shanghai Cancer Institute Renji Hospital Shanghai Jiaotong University School of Medicine Shanghai 200127 China

**Keywords:** non-alcoholic steatohepatitis, resident liver progenitor cell, macrophage, inflammation, fibrosis

## Abstract

Resident CD24 ^+^LCN2 ^+^ liver progenitor cells (LPCs) reportedly contribute to the expanding ductular reaction and macrophage-mediated inflammation associated with chronic liver damage. Both ductular reactions and macrophage-driven inflammation are associated with liver fibrosis and injury in various mouse liver disorders. This study aims to investigate the molecular phenotypes of LPCs and their regulatory mechanisms in humans with non-alcoholic steatohepatitis (NASH). Single-cell RNA sequencing (scRNA-seq) datasets are used to characterize the status and molecular phenotypes of LPCs in clinical NASH samples. To elucidate the regulatory mechanisms of LPCs, CellChat and NicheNet are employed to assess cell-cell communication between LPCs and other cell types. The findings are validated using RNA sequencing datasets associated with NASH progression, NASH mouse models (CDAHFD and HFD), and human NASH liver samples. Results show that resident CD24 ^+^LCN2 ^+^ LPCs are identified and found to be significantly enriched in NASH patients. Cell communication analyses predict strong interactions between LPCs and proinflammatory macrophage subtypes. Additionally, in NASH, the liver recruits peripheral blood mononuclear cell (PBMC)-derived macrophages and polarizes them into proinflammatory subtypes. The macrophage subtype MP-2 is identified as the primary recipient of LPC-derived signals, exhibiting marked hyperactivation of the NF-κB pathway and a strong association with liver fibrosis. Finally, the MP-2 markers COL10A1 and TPPP3 are characterized and validated. In summary, this study reveals that resident CD24 ^+^LCN2 ^+^ LPCs are activated in NASH and contribute to fibrosis progression by promoting the activation of the proinflammatory COL10A1 ^+^ TPPP3 ^+^ macrophage subtype.

## Introduction

Metabolic dysfunction-associated steatotic liver disease (MASLD) is an increasingly recognized liver disease worldwide, with a reported incidence of approximately 20%–30% [ 1, 2]. MASLD comprises a continuum of liver conditions, ranging from benign metabolic dysfunction-associated steatosis to its progressive form characterized by inflammation and fibrosis, known as non-alcoholic steatohepatitis (NASH). The prevalence of NASH is high, and it poses a significant risk of progression to cirrhosis and hepatocellular carcinoma (HCC) [ 3, 4]. Current guidelines recommend lifestyle modifications and a limited number of MASLD-specific pharmacological treatments, such as vitamin E and pioglitazone, for managing the disease [4]. However, there is no effective strategy for reversing NASH or improving fibrosis. Therefore, further research is needed to elucidate the underlying mechanisms of NASH and explore potential innovative therapeutic approaches.

Recent studies have demonstrated that the ductular reaction is associated with liver fibrosis and damage in various liver disorders, including NASH. This process is often accompanied by the activation of liver progenitor cells (LPCs), which express markers related to biliary epithelial cells and exhibit proliferative and inflammatory responses [ 5, 6]. Our previous studies identified CD24 ^+^LCN2 ^+^ LPCs as the predominant resident LPCs contributing to the expanding ductular reaction and macrophage-mediated inflammation in chronic liver injury [7]. Emerging evidence suggests that crosstalk between resident LPCs and infiltrating immune cells promotes fibrogenesis through macrophage-derived profibrotic mediators, including TNF-α, NF-κB, and TGF-β [ 8– 12]. This fibrotic reprogramming represents a key driver of NASH pathogenesis, as fibrosis is the strongest histological predictor of adverse clinical outcomes, including cirrhosis progression, transplant-free survival, and liver-related mortality. However, the mechanism by which LPC-activated inflammatory responses promote the pathogenesis of NASH remains unclear. Therefore, understanding the interactions between liver intrinsic LPCs and inflammatory cells as well as their impact on fibrosis is critical and may help reveal the pathological mechanisms of NASH. This may further lead to the development of new therapeutic strategies.

In the present study, we aimed to explore the molecular phenotypes of LPCs and their regulatory mechanisms in NASH. We found that resident LCN2 ^+^CD24 ^+^ LPCs were activated in NASH and that the macrophage subset closely communicating with resident LCN2 ^+^CD24 ^+^ LPCs highly expressed the fibrosis-related genes tubulin polymerization promoting protein family member 3 ( *TPPP3*) and collagen type X alpha 1 chain ( *COL10A1*). These findings confirm the mechanism by which LCN2 ^+^CD24 ^+^ LPCs activate macrophages to promote NASH and contribute to advances in LPC-based treatments for NASH.

## Materials and Methods

### Data acquisition for NASH

To evaluate the changes in various cell populations in NASH patients compared with healthy individuals, single-cell transcriptomic datasets were downloaded from the Gene Expression Omnibus (GEO) database ( https://www.ncbi.nlm.nih.gov/). The datasets included transcriptomic data from three healthy groups (two samples of healthy livers: GSM4041150--GSM4041152, GSM4041158--GSM4041159; one sample of healthy PBMCs: GSM7818498) and three MASLD-cirrhotic patients (two samples of MASLD-cirrhotic livers: GSM4041161--GSM4041163, GSM4041168--GSM4041172; one sample of MASLD-cirrhotic PBMCs: GSM4041170). For the validation study, RNA sequencing data from the GSE135251 and GSE240729 datasets were analyzed, comprising 283 liver biopsy samples, including 10 healthy controls and 273 individuals with MASLD.

### Single-cell RNA sequencing analysis

The single-cell RNA sequencing (scRNA-seq) data analysis was performed using the “Seurat” package (version 4.4.0) [13]. The genes that were expressed in fewer than three cells within a sample were excluded. The same criteria was applied to cells that had fewer than 300 detected genes or whose total mitochondrial gene content exceeded 30%. All the datasets were normalized using standard log normalization. A total of 2000 highly variable genes were identified for subsequent principal component analysis (PCA). The clusters were identified using the Seurat function FindClusters, visualized via uniform manifold approximation and projection (UMAP) analysis, and then annotated on the basis of the expression of known cell markers. Special markers for each cluster were identified via the Wilcoxon rank sum test with Bonferroni correction, as implemented in the FindMarkers function. Genes with an expression difference of at least 0.25 on a natural log scale and a difference of at least 0.25 in the percentage of detected cells, with an adjusted *P* value less than 0.05, were considered significant.

### Analysis of cell-cell communication and protein interaction networks

A systematic analysis of cell-cell communication was performed using the CellChat R package (v1.6.1) [14] and NicheNet (v2.2.0) [ 15, 16 ]. The standard CellChat workflow was employed to analyze cell-cell communication and predict the primary signaling inputs and outputs of cells. CellChat, with its comprehensive ligand-receptor interaction database and network-based analytical approach, provides a global perspective on cell-cell communication patterns within the dataset. In parallel, NicheNet was utilized to infer ligand-receptor-mediated cell-cell communication. By integrating ligand-receptor interaction networks and gene regulatory networks, NicheNet predicts how sending cells influence the gene expression of receiving cells through ligand secretion. Specifically, NicheNet (v1.1.0) was used to identify ligand-receptor interactions that regulate LPC-MP-2 interplay in NASH. The STRING database was used for a preliminary analysis of protein-protein interaction (PPI) networks involving molecular markers and key ligand-receptor pairs associated with cell-cell communication between LPCs and the MP-2 subtype.

### Functional enrichment analysis

The Kyoto Encyclopedia of Genes and Genomes (KEGG) [17] was used to explore the potential biological functions associated with cluster-specific markers. The analysis was conducted via the R package clusterProfiler (version 4.10.0) [ 18, 19]. Additionally, QuSAGE (version 2.36.0) [20] analysis was performed to characterize the relative activation of specific gene sets, including proinflammatory markers and the NF-κB signaling pathway.

### Verification of candidate markers during NASH development

The RNA-seq datasets from GSE135251 and GSE240729 were used to validate the significance of the identified candidate markers in NASH development. Processed and annotated count data were obtained from the GEO database, and differential expression analysis was performed using the DESeq2 (v1.42.1) package [21]. Genes with an absolute log (fold change) greater than 1 and an adjusted *P* value of less than 0.05 were considered statistically significant.

### Experimental animals

Healthy male C57BL/6J mice (6–8 weeks old, 18–26 g) were purchased from Nanjing Viton Lihua Co. (Nanjing, China). All the mice were housed in a specific pathogen-free (SPF) environment under a 12/12-h light/dark cycle at a temperature of 22 ± 2°C and a relative humidity of 50% ± 5%. They were provided ad libitum access to water and standard chow for 14 days to acclimate before the experiment. All animal experiments were conducted in compliance with ethical guidelines. The procedures adhered to the Reporting of *In Vivo* Experiments Guidelines for the Care and Use of Laboratory Animals and were approved by the Institutional Animal Care and Use Committee of Shanghai Model Organisms Center Inc. (IACUC2019-0027-06).

### Diet-induced NASH model

C57BL/6J mice were randomly assigned to three groups ( *n* = 6/group): (1) the CDAHFD group-fed with a choline-deficient, L-amino acid-defined high-fat diet (CDAHFD; 25% fat, olive oil/beef tallow) for 6‒8 weeks to induce MASLD via impaired lipid transport and steatosis [22]; (2) the HFD group-fed with a high-fat diet (HFD; 60% fat, lard/soybean oil) for 12‒24 weeks, leading to lipid overload and insulin resistance [ 23– 25]; and (3) the normal diet control group-fed with a standard diet. Both the CDAHFD (choline/amino acid deficiency) and HFD models promote NASH progression through steatosis, inflammation, and fibrosis. Following euthanasia, liver and adipose tissues were collected, weighed, and processed for histological analysis (fixed in 4% paraformaldehyde) or molecular analysis (snap-frozen at –80°C) [ 22– 25].

### Human liver biopsies

Liver biopsy samples from patients with NASH were obtained from Renji Hospital, School of Medicine, Shanghai Jiao Tong University, a leading medical center specializing in liver disease diagnosis and treatment. This human tissue research was approved by the Human Research Ethics Committee of Renji Hospital (Ethics Approval Number: KY2024-117-B). All procedures involving human tissue samples complied with the ethical standards of the Declaration of Helsinki and its subsequent amendments. Informed consent was obtained from all participants before sample collection, ensuring a full understanding of the study’s purpose, methods, and potential implications. The confidentiality of personal information associated with the samples was strictly maintained ( Supplementary Table S1). Liver tissue samples were obtained via percutaneous liver biopsy under local anesthesia performed by experienced hepatologists using ultrasound guidance. After collection, the tissue was divided into two portions: one fixed in 10% neutral-buffered formalin for histological examination and the other rapidly frozen in liquid nitrogen and stored at –80°C for molecular analysis.

### H&E staining and immunohistochemistry

Paraffin-embedded mouse and human liver tissues were sectioned into 5-μm thick slices and stained with hematoxylin and eosin (H&E) to assess morphological changes. Immunohistochemical staining was performed to detect the expressions of collagen 10A1 and TPPP3 in mouse and human liver tissues. The following primary antibodies were used: anti-collagen 10A1 antibody (1:1000; 15057-1-AP; Proteintech, Chicago, USA), anti-β-actin antibody (1:1000; #4970; Cell Signaling Technology, Boston, USA) and anti-TPPP3 antibody (1:1000; #72026; Cell Signaling Technology). Detailed antibody information is provided in Supplementary Table S2. Paraffin sections were deparaffinized using xylene, followed by sequential rehydration in anhydrous ethanol, 95% ethanol, and 70% ethanol. Antigen retrieval was performed using a hydrogen peroxide blocking solution at room temperature. The sections were then incubated with 5% bovine serum albumin (BSA) blocking solution, followed by overnight incubation with the primary antibody at 4°C. After washing, the sections were treated with the appropriate secondary antibody and counterstained with hematoxylin. The staining intensity was quantified using ImageJ software (National Institutes of Health, Bethesda, USA).

### Real-time quantitative PCR (RT-qPCR)

RT-qPCR was used to assess gene expression levels. Total RNA was extracted from liver tissues using TRIzol reagent (Life Technologies, Carlsbad, USA) following the manufacturer’s protocol. The RNA transcripts were quantified via the Brilliant SYBR Green qPCR kit (TaKaRa, Kyoto, Japan) on a Roche LightCycler 4800 II real-time PCR system (Roche, Basel, Switzerland). The specific primers used for amplification are detailed in Supplementary Tables S3 and S4. *β-Actin* was used as an internal reference, and relative gene expression levels were calculated using the 2 ^–ΔΔCt^ method to compare expression differences among samples.

### Western blot analysis

Liver samples were harvested and lysed in RIPA buffer supplemented with PMSF and a protease inhibitor cocktail. The lysates were mixed by inversion at 4°C for 1 h, followed by centrifugation at 12,000 *g* at 4°C for 15 min. The supernatants were collected for protein concentration measurement using a BCA kit (Thermo Fisher Scientific, Rockford, USA). Proteins were separated by SDS-PAGE, transferred onto PVDF membranes (Hybond-P), and blocked in 5% nonfat milk in Tween-20 Tris-buffered saline for 1 h. The membranes were incubated with primary antibodies overnight at 4°C, followed by incubation with HRP-conjugated secondary antibodies for 1 h at room temperature. The protein bands were visualized using an enhanced chemiluminescence kit (Thermo Fisher Scientific) and detected via a ChemiDoc™ XRS+ system (Bio-Rad, Hercules, USA). The antibody details are provided in Supplementary Table S2.

### Statistical analysis

Fisher’s exact test was used to compare the proportions of LPCs in cholangiocyte subgroups between healthy and NASH liver samples. Pearson’s correlation analysis was performed to evaluate gene expression patterns during NASH development. For missing data, the multiple imputation by chained equations (MICE) method was employed to estimate missing values on the basis of dataset relationships. All experimental data were derived from three independent biological replicates with triplicate technical measurements, unless otherwise specified. Statistical analyses were conducted via GraphPad Prism software (v10.4; GraphPad Software, Inc, La Jolla, USA). The quantitative results are expressed as the mean ± standard deviation (SD). For comparisons between two groups, unpaired two-tailed Student’s *t* tests were applied. Multiple group analyses were performed using one-way ANOVA followed by Bonferroni post hoc correction for multiple comparisons. *P* < 0.05 was considered statistically significant for all analyses.

## Results

### Identification of a subset of LCN2 ^+^CD24 ^+^ LPCs in MASLD

Four single-cell transcriptome liver tissue samples from healthy individuals and MASLD-cirrhotic patients were collected to investigate the transcriptome changes associated with MASLD. After rigorous quality control, 11,295 and 10,555 cells were obtained from the transcriptome data of healthy and MASLD samples, respectively. In both the healthy and MASLD samples, the cells were divided into 12 clusters ( Supplementary Figure S1A) using classical lineage-specific markers, including T cells (IL7R and TNF), innate lymphoid cells (ILCs) (KLRC1, PRF1, and FGFBP2), cholangiocytes (ANXA4 and CFTR), macrophages (MSR1, CYBB, and CD163), endothelial cells (KDR, ENG, ADGRF5, and EGFL7), B cells (CD79A and MS4A1), hepatic stellate cells (HSCs) (COL1A2, CCL19, DCN, and HGF), plasma cells (JCHAIN, IGKC, IGHG3, and JSRP1), cycling cells (MKI67, TOP2A, UBE2C, and RRM2), plasmacytoid dendritic cells (pDCs) (LILRA4, LRRC26, and CLEC4C), epithelial cells (ALB, F2, CYP4A11, and APOC2), and mast cells (TPSAB1 and CPA3) ( Supplementary Figure S1B). The frequency of cell types varied between the healthy and MASLD liver samples ( Supplementary Figure S1C). A higher population of cholangiocytes and epithelial cells was observed in the MASLD liver, with a frequency of more than 75%, than in the healthy liver. This could be attributed to the occurrence of the ductular reaction in MASLD.

**Figure 1:**
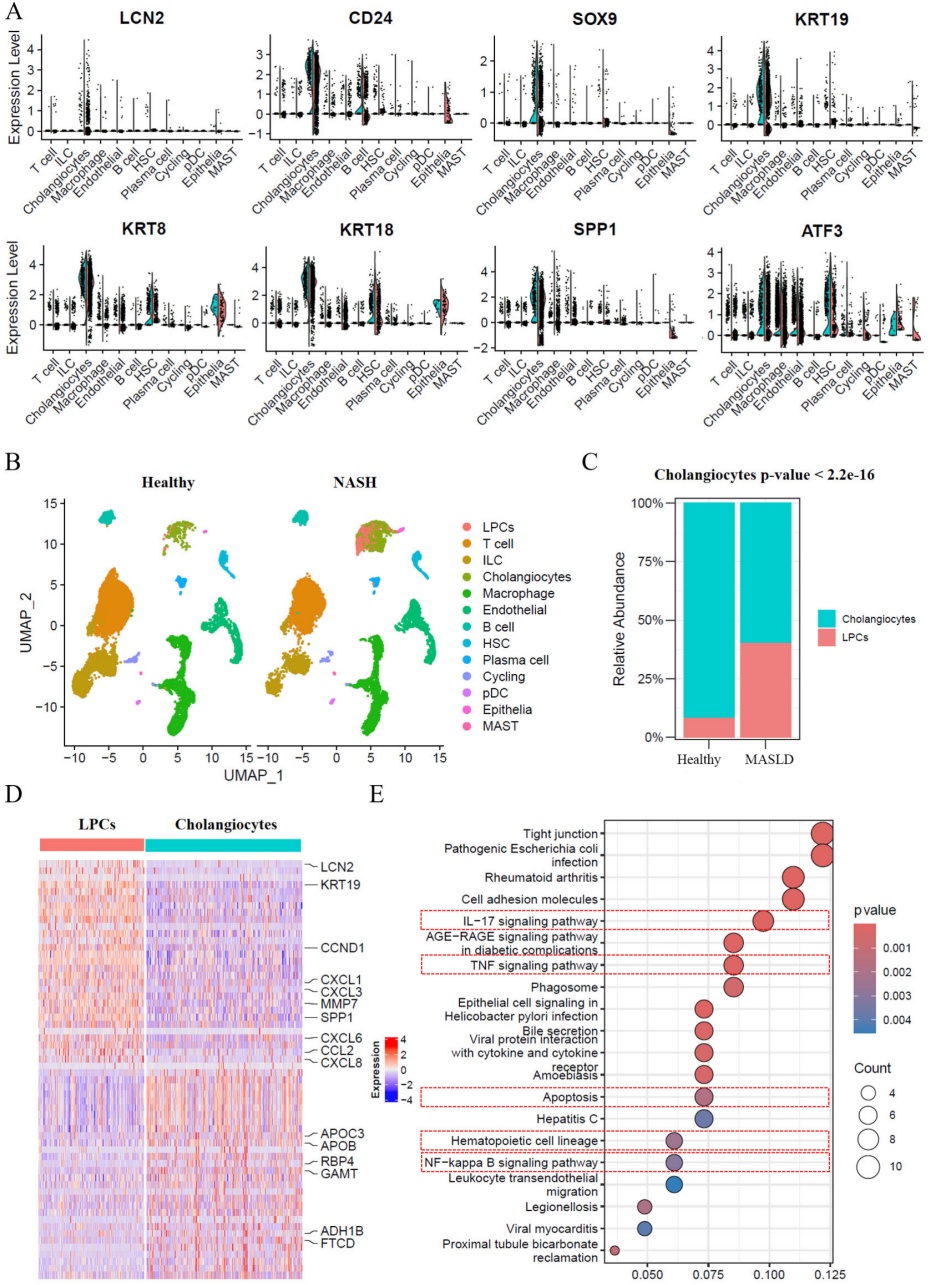
Figure 1 Increased LCN2 ^+^CD24 ^+^ LPCs are identified in the cholangiocyte subset of NASH livers (A) The violin plot shows the expression levels of LCN2, CD24, SOX9, and KRT19 in nonparenchymal cells of the human liver. (B) UMAP visualization revealed the distribution of LCN2+ CD24+ LPCs within the cholangiocyte subset. (C) Compared with that in healthy livers, a significant increase in the proportion of LPCs in the cholangiocyte subset occurred in NASH livers. (D) Heatmap of the top 30 highly expressed genes in LPCs compared with cholangiocytes. (E) The top 20 enriched KEGG pathways of highly expressed genes in LPCs compared with cholangiocytes included the IL-17 signaling pathway, TNFα inflammatory pathway, NF-κB signaling pathway, cell proliferation and apoptosis, and hematopoietic cell recruitment and differentiation.

Our previous studies identified CD24 ^+^LCN2 ^+^ LPCs as the major resident LPCs that contribute to the expanding ductular reaction in chronic liver damage [7]. The expression of reported progenitor gene markers in each cell subgroup was observed to investigate the characteristics and existence of resident LPCs in MASLD ( Figure 1A). The cholangiocytes showed higher expressions of CD24 and LCN2, which are LPC markers. Other precursor genes, such as *SOX9*, *KRT19*, *KRT8*, *KRT18*, *SPP1*, and *ATF13*, were also highly expressed in the subpopulation of cholangiocytes. The CD24 ^+^LCN2 ^+^SOX9 ^+^KRT19 ^+^ cells were identified as LPCs and were significantly enriched in the subpopulation of cholangiocytes ( Figure 1B,C). These results suggest that in MASLD, ductal reaction cells are composed primarily of liver-resident LPCs.

To analyze the molecular characteristics of the resident LPCs defined in our study, differential expression analysis between LPCs and cholangiocytes was performed. The inflammatory genes (including *CCND1*, *MMP7*, *CCL2*, *CXCL1*, *CXCL3*, CXCL6, and *CXCL8*) and precursor genes (such as *LCN2* , *KRT19*, and *SPP1*) were highly expressed in LPCs ( Figure 1D). The findings from the KEGG enrichment analysis further revealed that highly expressed genes in LPCs were enriched mainly in immune-related pathways. These pathways included immune system pathways (IL-17 signaling pathway and hematopoietic cell lineage), inflammatory pathways (TNF signaling pathway and NF-κB signaling pathway) and apoptosis pathways ( Figure 1E). These results suggested that in MASLD, the subpopulation of LCN2 ^+^CD24 ^+^ LPCs significantly increased, highlighting their possible participation in liver cell damage repair and inflammatory responses. Consistent with these findings, several studies have reported that in MASLD, LPCs secrete chemokines and proinflammatory mediators during the ductular reaction, contributing to fibrosis progression [ 26– 28].

### Assessment of the relationship between LCN2 ^+^CD24 ^+^ LPCs and proinflammatory macrophages

**Figure 2:**
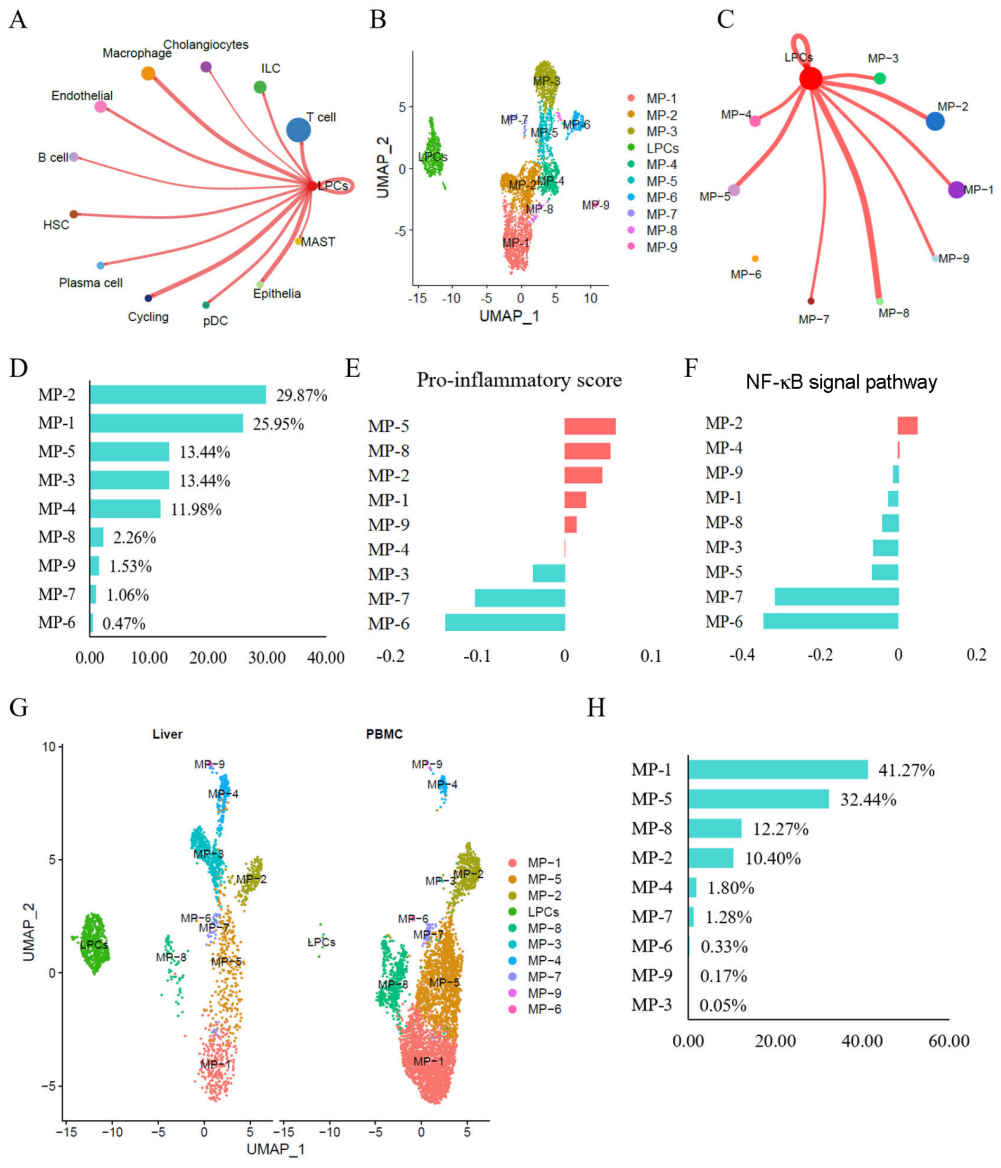
Figure 2 LCN2 ^+^CD24 ^+^ LPCs are closely associated with proinflammatory macrophages in NASH livers (A) Summary of the absolute number of probable cell-cell interaction pathways between LCN2+ CD24+ LPCs and other cell subpopulations in NASH livers. (B) UMAP plot of resident LPCs and macrophage subsets in healthy and NASH livers. (C) Number of interactions illustrating cell-cell communication between LPCs and different macrophage subsets in MASLD livers. (D) Distribution proportions of macrophage subtypes in NASH livers. (E) QuSAGE (version 2.28.0, KEGG database) analysis was used to quantify proinflammatory marker expression across different macrophage clusters. (F) QuSAGE analysis (version 2.28.0, KEGG database) was used to assess the activation rate of the NF-κB signaling pathway in different macrophage clusters. (G) UMAP visualization of macrophage subsets recruited from PBMCs via MASLD. (H) Proportion of each macrophage subset recruited from PBMCs in MASLD.

Cell-cell interaction analyses of LPCs and immune-related cells were performed to decipher the ligand-receptor interactions associated with MASLD. The results from CellChat indicated that in MASLD, LPCs had the strongest interaction with macrophages, as evidenced by the relatively high number of interactions ( Figure 2A). While macrophages are well-established regulators of hepatic immune homeostasis and lipid metabolism [ 29– 32], their functional plasticity and phenotypic diversity during MASLD progression remain incompletely characterized. To systematically map the dynamic reprogramming of macrophage subtypes from healthy to MASLD, macrophages were re-clustered and divided into nine subpopulations on the basis of known lineage markers ( Figure 2B and Supplementary Figure S2). Analysis of cell-cell communication between LPCs and macrophage subtypes revealed robust interactions between LPCs and three proinflammatory macrophage subpopulations: MP-5, MP-8, and MP-2 ( Figure 2C,E). Among these subtypes, MP-2 emerged as the predominant subtype ( Figure 2D). Notably, the NF-κB signaling pathway, previously implicated in fibrosis promotion in NASH [33], was significantly activated in MP-2 ( Figure 2F). These findings suggest that MP-2 may play a critical role in driving inflammatory and fibrotic responses within the hepatic microenvironment, underscoring its potential as a therapeutic target in NASH progression.

The proportions of macrophage subtypes were lower in the MASLD liver samples than in the healthy samples ( Supplementary Figure S1C). Studies have shown that when liver injury occurs, peripheral blood-derived macrophages accumulate in the liver to replenish the lost macrophages and release inflammatory and fibrotic factors [ 34, 35]. Therefore, scRNA-seq datasets of healthy (GSM7818497–GSM7818498) and MASLD (GSM4041170, GSM4041172, and GSM4041173) peripheral blood mononuclear cells (PBMCs) were used to analyze the recruitment of macrophages into the liver. Using known lineage marker signatures, the PBMC samples from the healthy and MASLD groups were clustered and annotated ( Supplementary Figure S3A,B). After the use of the UMAP method for dimensionality reduction and visualization of the clustering results, the proportion of macrophage subpopulations in PBMCs was significantly increased in the MASLD samples than in the healthy samples ( Supplementary Figure S3C). Further analysis of the types of recruited macrophages revealed that more than 90% were proinflammatory macrophages, including MP-1, MP-2, MP-5, and MP-8 ( Figure 2G,H). These results indicate that during NASH, the liver recruits PBMCs and polarizes them into M1 proinflammatory subpopulations, thereby participating in hepatic immune homeostasis.

### A subset of proinflammatory TPPP3 ^+^COL10A1 ^+^ macrophages is found to be involved in regulating the fibrotic process of MASLD

**Figure 3:**
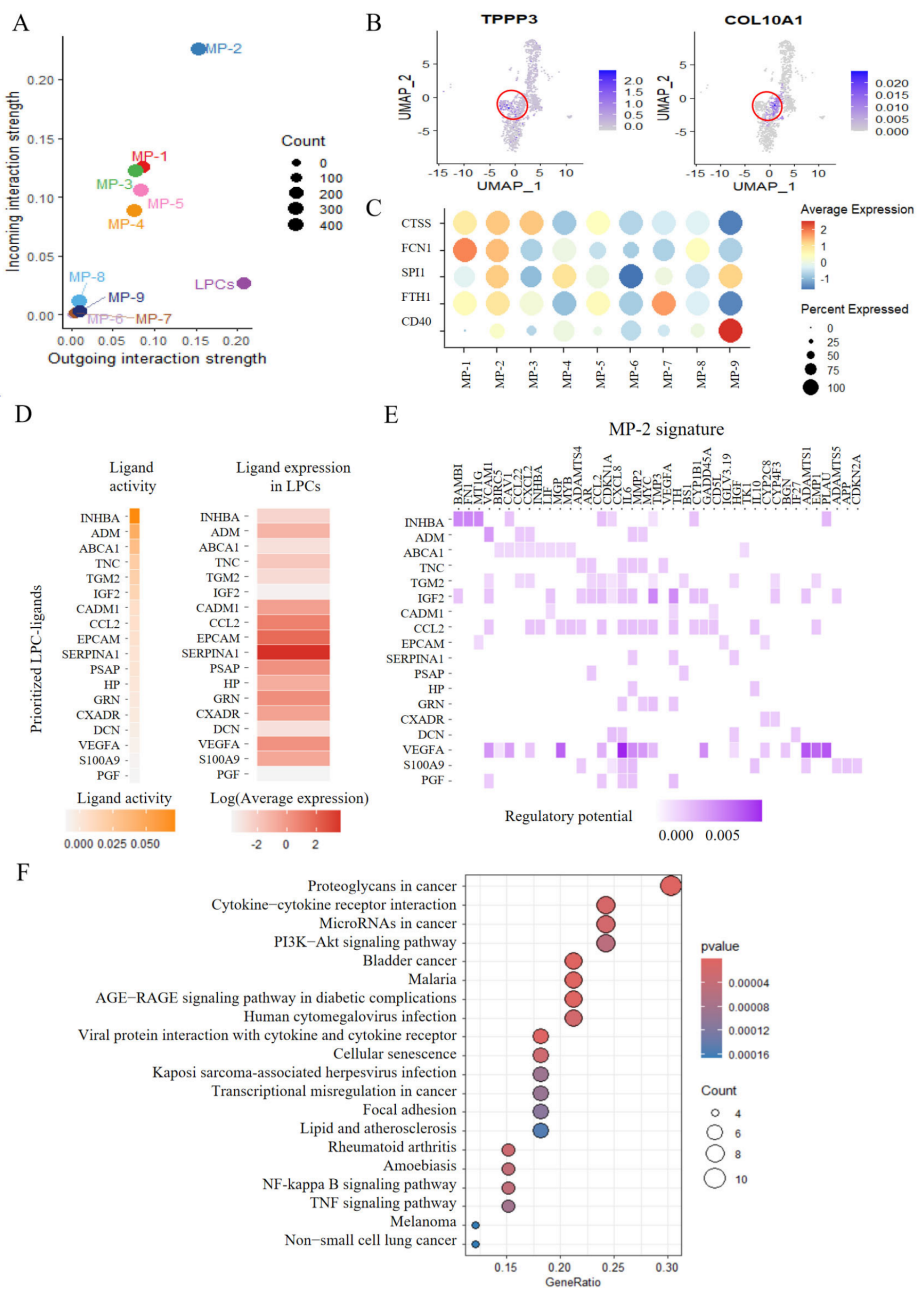
Figure 3 Strong cell-cell communication occurs between LPCs and MP-2 macrophages (A) Two-dimensional spatial analysis using CellChat revealed LPCs as primary signal senders and MP-2 macrophages as primary signal receivers. (B) Expressions of two fibrosis-related genes in MP-2 macrophages. (C) Expressions of additional fibrosis-related genes across different macrophage subtypes in MASLD. (D) NicheNet analysis validating key ligands in LPCs and their expression levels. The left panel shows a bar plot of the predicted ligand activity scores, with higher scores indicating stronger regulatory potential. The right panel displays a Heatmap of the average expression levels (log2-transformed) of these ligands in LPCs, where red indicates increased expression. Ligands are ranked by their activity scores. (E) NicheNet analysis identifying target genes in MP-2 macrophages regulated by key LPC ligands. (F) Top 20 enriched KEGG pathways associated with the MP-2 macrophage targets identified in (E).

To further investigate the molecular mechanisms by which LPCs regulate macrophages, outgoing and incoming signals in MASLD were reviewed ( Supplementary Figure S4B). The results showed that MP-2 is the main receiver of the signals emitted by LPCs ( Figure 3A). The fibrosis genes *TPPP3* and C *OL10A1* were highly expressed in MP-2 ( Figure 3B and Supplementary Figure S4A). In addition, the fibrosis-associated genes *CTSS* , *FCN1*, *SPI1*, *FTH1*, and *CD40* were highly expressed in MP-2 ( Figure 3C). Additionally, LPCs and MP-2 macrophages strongly interact in inflammation-related signaling pathways, including MIF, COMPLEMENT, ANGPTL, NOTCH, JAM, and CX3C ( Supplementary Figure S4B). These results suggest that the proinflammatory MP-2 subpopulation is a key driver of MASLD-related fibrogenesis.

The NicheNet package was subsequently employed to analyze LPC-MP-2 communication further ( Figure 3D–F). This analysis identified key ligands expressed by LPCs that are predicted to regulate target genes in MP-2 macrophages. Notably, these target genes were enriched in inflammation- and fibrosis-related pathways, which was consistent with the results of the CellChat analysis ( Supplementary Figure S4B) and the QuSAGE analysis of NF-κB signaling pathway activation ( Figure 2F). These analytical results further reinforce the robustness and reliability of our conclusions.

### Validation of fibrosis markers in proinflammatory macrophages

**Figure 4:**
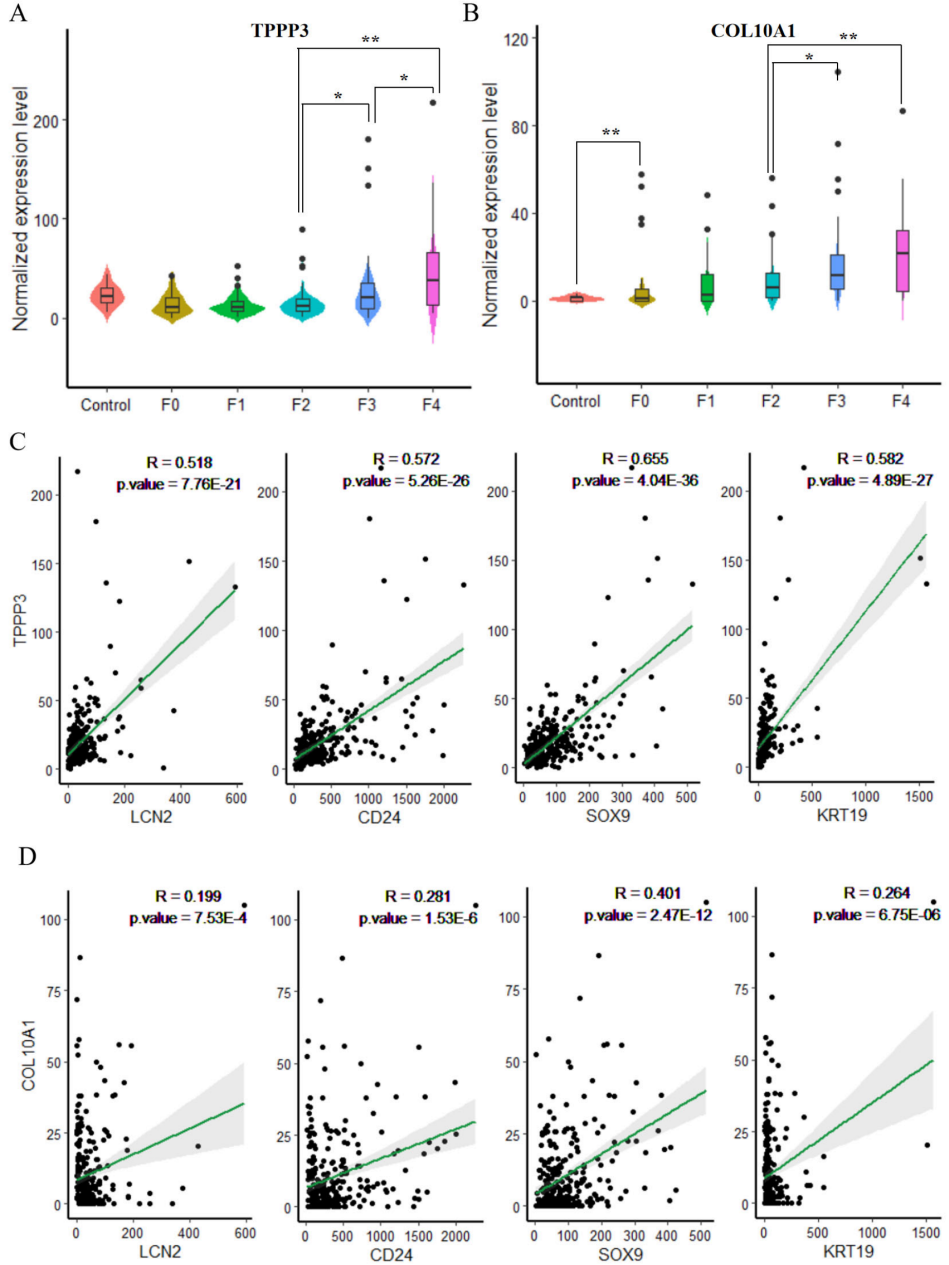
Figure 4 Validation of the fibrosis-related genes *TPPP3* and *COL10A1* in bulk RNA-seq datasets (A) The boxplot shows the differences in the expression of the TPPP3 gene at different stages of NASH development. Compared with that in F2, expression was significantly greater in F3 and significantly higher in F4 than in F3 [* P < 0.05, log (fold change) < 2; **P < 0.01, log (fold change) ≥ 2]. (B) The boxplot shows the expression differences of the COL10A1 gene at different stages of NASH development. Compared with that in F0 and F1, expression in F3 and F4 was significantly greater than that in F2 [*P < 0.05, log (fold change) < 2; **P < 0.01, log (fold change) ≥ 2]. (C) The relationships between the expression of TPPP3 and the expressions of marker genes of LPCs were measured via Pearson’s product-moment correlation analysis. (D) The relationship between the expression of COL10A1 and the expressions of marker genes of LPCs was measured via Pearson’s product-moment correlation analysis.

These studies suggest that the proinflammatory MP-2 subpopulation plays a role in regulating the fibrotic process in NASH. However, the correlation between the expression levels of TPPP3 and COL10A1 in MP-2 and the progression of NASH-related fibrosis remains unclear. To validate the expressions of the two highly expressed genes at different stages of MASLD development and progression, two RNA-seq datasets (GSE135251 and GSE240729) were integrated. A total of 283 samples were included in this validation analysis and classified on the basis of fibrosis progression: 10 normal control samples, 47 F0-stage samples, 64 F1-stage samples, 78 F2-stage samples, 64 F3-stage samples, and 20 F4-stage samples. Differential gene expression analysis revealed significant upregulation of *TPPP3* and *COL10A1* in the F3 and F4 stages ( Figure 4A,B). The expression levels of *TPPP3* and *COL10A2* increased as the degree of liver fibrosis increased. Pearson’s correlation analysis revealed that the expressions of TPPP3 and COL10A1 was positively correlated with the expressions of CD24, LCN2, SOX9, and KRT19, all of which are LPC-specific molecular markers ( Figure 4C,D). These results suggest that CD24 ^+^LCN2 ^+^ LPCs might regulate the TPPP3 ^+^COL10A1 ^+^ proinflammatory MP-2 subpopulation involved in the profibrotic progression process of MASLD.

In addition, the STRING database was used to perform a preliminary analysis of the PPI networks among the molecular markers of LPCs, the molecular markers of MP-2, and the key ligands and their target genes that regulate MP-2 in LPCs, as identified through NicheNet analysis ( Supplementary Figure S5). This network highlights potential interactions among proteins encoded by these genes. The results revealed that the LPC molecular markers CD24 and LCN2 regulate COL10A1 through the ligand-receptor interaction network in the LPC-MP-2 axis. These genes are functionally associated with fibrosis-related pathways, including the interleukin-4 and interleukin-13 signaling pathways, as well as extracellular matrix organization. However, the interaction between these markers and TPPP3 requires further experimental validation. PPI network analysis further demonstrated a functional interaction between LPCs and the MP-2 subtype.

### TPPP3 and COL10A1 expression is significantly increased in advanced fibrosis stages in MASLD model mice and human clinical samples

**Figure 5:**
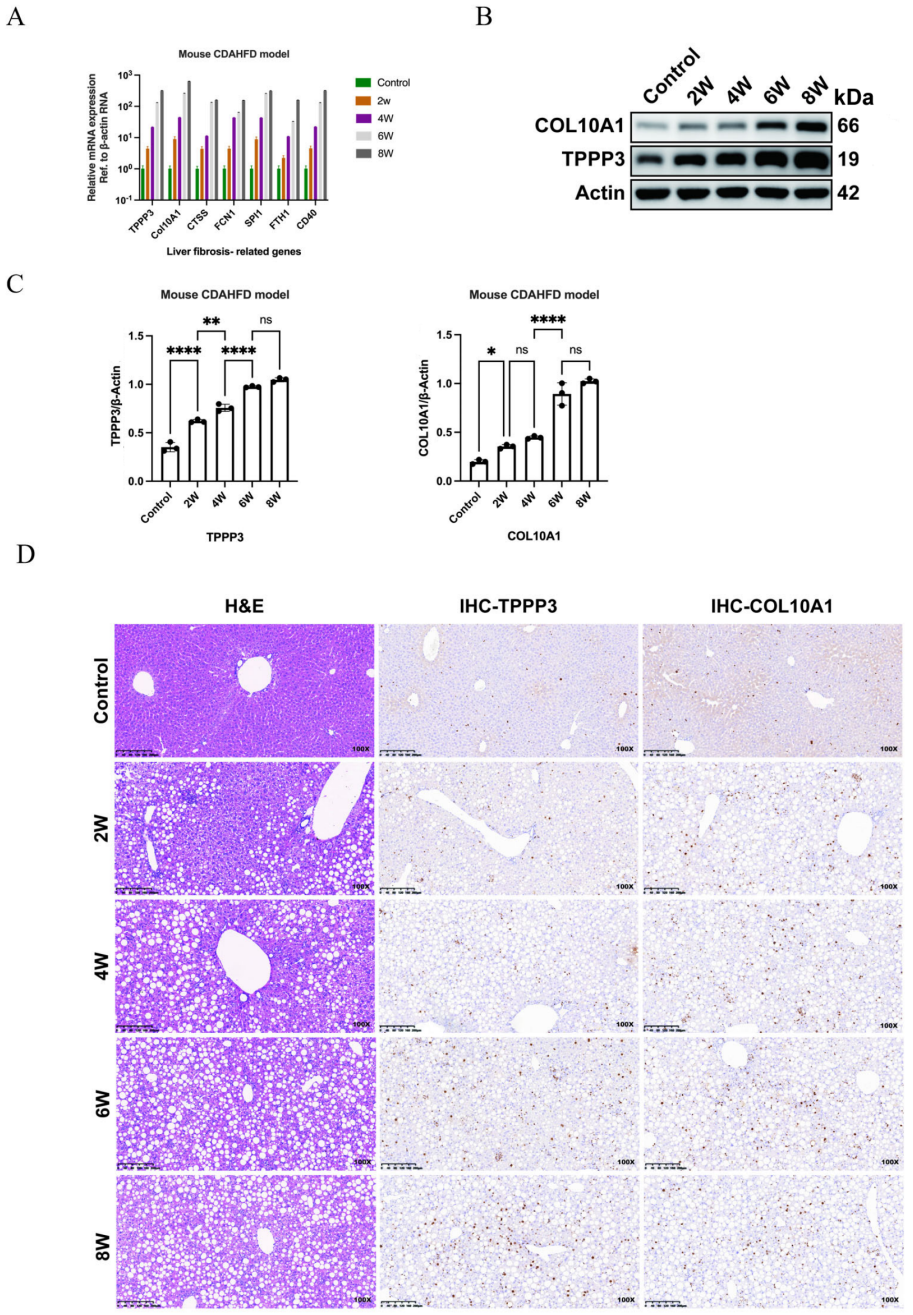
Figure 5 Expressions of TPPP3 and COL10A1 are significantly increased in areas of advanced fibrosis in the liver tissue of CDAHFD-induced NASH mice (A) qPCR results showing the expressions of fibrosis-related genes in CDAHFD-induced NASH model mice fed with a CDAHFD for various durations (in weeks). (B) Western blot analysis results for TPPP3 and COL10A1 expressions in CDAHFD-induced NASH model mice fed with a CDAHFD for various durations (in weeks). β-Actin was utilized as a loading control for accurate protein quantification and normalization. (C) Summarized western blot data showing the relative protein levels of TPPP3 and COL10A1 in CDAHFD-induced NASH mice fed with a CDAHFD for various durations (in weeks). “ns” indicates no statistical significance; *P < 0.05, **P < 0.01, and ****P < 0.0001. (D) Immunohistochemistry images showing TPPP3 and COL10A1 expression in CDAHFD-induced NASH mice fed with a CDAHFD for various durations (in weeks). Scale bar: 200 μm. The magnification of the picture is 100×.

To further validate these findings *in vivo*, we examined the expressions of TPPP3 and COL10A1 in liver tissues from NASH model mice, including CDAHFD-fed and HFD-fed mice. Fibrosis progression in these models was assessed on the basis of feeding duration and tissue staining. Histopathological evaluation via H&E staining demonstrated progressive hepatic pathology in both models. We next analyzed the expressions of the fibrosis-related genes *TPPP3* and *COL10A1* at different NASH stages using immunohistochemistry, qPCR, and western blot analysis. Gene expression profiling revealed elevated transcription levels of these pro-fibrotic markers in hepatic macrophages (MP-2 subset), paralleling the upregulation of fibrosis-associated genes ( *CTSS*, *FCN1*, *SPI1*, *FTH1*, and *CD40*) during disease progression ( Figure 5A and Supplementary Figure S6A). Western blot analysis confirmed the progressive accumulation of TPPP3 and COL10A1 proteins, with expression intensities correlating positively with fibrosis severity ( Figure 5B,C and Supplementary Figure S6B,C). Immunohistochemical staining further revealed increased expressions of COL10A1 and TPPP3 in fibrotic niches in advanced NASH tissues, which is consistent with their putative roles in extracellular matrix (ECM) remodeling and macrophage-mediated fibrogenesis ( Figure 5D and Supplementary Figure S6D). These experimental findings robustly validated the cellular dynamics predicted by single-cell transcriptomic profiling, establishing a mechanistic link between macrophage-derived molecular signatures and MASLD progression. Notably, the spatiotemporal upregulation of TPPP3 and COL10A1, key fibrogenic markers identified in MP-2 macrophages, exhibited striking concordance with histopathological fibrosis staging across both the CDAHFD and HFD models ( Figure 5 and Supplementary Figure S6). This expression trajectory, corroborated by orthogonal methods (qPCR, western blot analysis, and immunohistochemistry), was predominantly localized to peri-sinusoidal and bridging fibrotic regions, suggesting a direct role in ECM remodeling and macrophage-to-myofibroblast crosstalk. On the basis of emerging insights from single-cell analyses, we propose that TPPP3, a microtubule-associated protein involved in cytoskeletal reorganization, and COL10A1, non-fibrillar collagen enriched in fibrotic niches, coordinately regulate late-stage fibrogenesis through synergistic mechanisms. This regulatory axis promotes macrophage activation, induces ECM stiffening, and amplifies pro-fibrotic signaling pathways, particularly the TGF-β/Smad cascade. The progressive enrichment of these genes during NASH evolution ( Figure 5B–D and Supplementary Figure S6B–D) aligns with clinical observations of collagen subtype switching in advanced fibrosis, underscoring their potential as biomarkers or therapeutic targets for stage-specific interventions. These data not only confirm the predictive power of single-cell omics in mapping disease trajectories but also delineate a macrophage-driven molecular axis central to NASH-related fibrotic cascades, providing a framework for mechanistically informed therapeutic strategies.

**Figure 6:**
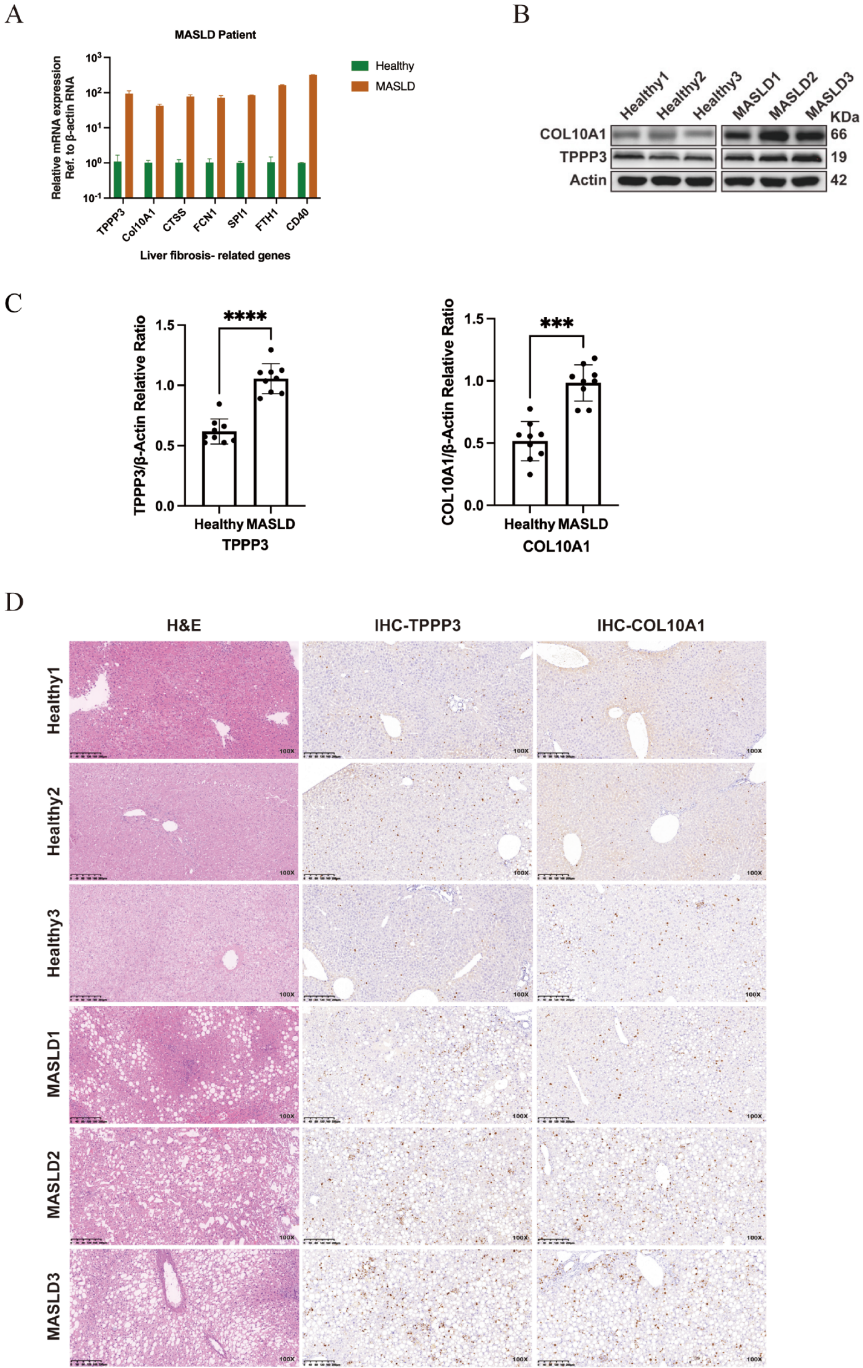
Figure 6 Expressions of TPPP3 and COL10A1 are elevated in MASLD patients (A) qPCR analysis of liver tissues from healthy controls and MASLD patients revealed increased mRNA expressions of fibrosis-related genes, including TPPP3 and COL10A1, in MASLD. (B) Western blot analysis of liver lysates from healthy controls and MASLD patients revealed increased protein levels of COL10A1 and TPPP3 in MASLD. β-Actin was utilized as a loading control for accurate protein quantification and normalization. (C) Quantification of TPPP3 and COL10A1 protein expression relative to that of β-actin via western blot analysis. “ns” indicates no statistical significance; ***P < 0.001, and ****P < 0.0001. (D) Representative images of H&E staining and immunohistochemistry staining for TPPP3 and COL10A1 in liver tissue sections from healthy controls and MASLD patients. Increased staining intensity for TPPP3 and COL10A1 is observed in MASLD tissues. Scale bar: 200 μm. The magnification of the picture is 100×.

Having established the spatiotemporal dynamics and mechanistic roles of TPPP3 and COL10A1 in murine NASH models, we next sought to determine whether these findings can translate to human MASLD pathogenesis. Liver tissues from healthy controls ( *n* = 3) and MASLD patients ( *n* = 3) were analyzed. qPCR analysis revealed significant upregulation of *TPPP3* and *COL10A1* mRNA in the MASLD cohort ( Figure 6A), a finding corroborated by western blot analysis, which revealed elevated protein expressions ( Figure 6B,C). Histopathological evaluation via H&E staining confirmed MASLD-associated steatosis and fibrosis, while IHC revealed that TPPP3 and COL10A1 localized to fibrotic septa and peri-sinusoidal regions, with staining intensities strongly correlated with fibrosis severity ( Figure 6D). These human data closely mirror the spatiotemporal dynamics observed in murine NASH models ( Figure 5), where TPPP3 and COL10A1 exhibited stage-specific enrichment in macrophage-rich fibrotic niches. The evolutionary conservation of their dysregulation—spanning transcriptional activation, protein accumulation, and spatial colocalization with ECM remodeling zones—suggests a fundamental role in fibrogenic cascades. Notably, the transition from COL1A1-dominated early fibrosis to COL10A1-enriched advanced stages (observed in both species) highlights a shared molecular axis driving collagen isoform switching. This interspecies consistency not only reinforces the biological plausibility of TPPP3/COL10A1 as core fibrogenic effectors but also positions them as translatable biomarkers for stratifying NASH progression or evaluating antifibrotic therapies.

## Discussion

This study elucidates the critical interplay between tissue-resident CD24 ^+^ LCN2 ^+^ LPCs and proinflammatory TPPP3 ^+^COL10A1 ^+^ macrophages in driving fibrotic progression in NASH. Through multiomics integration of scRNA-seq, histopathological evaluation, and transcriptional profiling of murine and human NASH tissues, we demonstrated that CD24 ^+^LCN2 ^+^ LPCs orchestrate ductular proliferation and TPPP3 ^+^COL10A1 ^+^ macrophage activation, thereby amplifying hepatic fibrogenesis through proinflammatory and fibrogenic crosstalk. These findings enhance our understanding of the cellular and molecular underpinnings of NASH fibrosis and identify novel therapeutic targets for intervention.

CD24 ^+^LCN2 ^+^ LPCs represent a distinct population of liver-resident progenitor cells essential for hepatic repair and regeneration. Under chronic injury conditions, these cells are activated and drive the ductular reaction, which is characterized by interlobular bile duct hyperplasia, stromal remodeling, and inflammatory infiltration [ 28, 36– 38]. scRNA-seq analysis revealed a significant enrichment of CD24 ^+^LCN2 ^+^ LPCs in NASH livers, with upregulated expression of genes associated with inflammation ( *e*. *g*., *IL6*, *CXCL1*) [28], fibrogenesis ( *e*. *g*., *TGFB1*, *CTGF*) [39], and proliferation ( *e* . *g*., *MKI67*) [40]. Pathway enrichment analysis further highlighted the activation of the IL-17 [34], TNF-α [41], and NF-κB signaling pathways in these cells [42]. Among these pathways, NF-κB (nuclear factor kappa-light-chain-enhancer of activated B cells) plays a pivotal role in linking NASH to inflammation, macrophage polarization, and fibrosis. As a key transcription factor, NF-κB regulates proinflammatory cytokines, including TNF-α, IL-6, and IL-1β. In NASH, hepatic fat accumulation and damage to non-parenchymal cells, including LPCs, trigger NF-κB activation, leading to macrophage recruitment and exacerbated inflammation. Notably, NF-κB signaling promotes the proliferation of M1 macrophages (proinflammatory) while suppressing M2 macrophages (anti-inflammatory), thereby exacerbating liver injury. Given its central role in disease progression, targeting the NF-κB pathway may represent a promising therapeutic strategy for mitigating inflammation and fibrosis in NASH, underscoring the need for further mechanistic investigations [ 43, 44].

While prior studies have individually examined the roles of LPCs and macrophages in NASH pathogenesis, their bidirectional crosstalk within the fibrotic niche remains poorly characterized. Furthermore, the specific interactions between LPCs and distinct macrophage subpopulations have not been well defined. Our study provides compelling evidence that CD24 ^+^LCN2 ^+^ LPCs engage in multifaceted communication with TPPP3 ^+^COL10A1 ^+^ macrophages through multiple inflammation-licensed signaling axes, including MIF [45], COMPLEMENT [46], ANGPTL [47], NOTCH [48], JAM [49], and CX3C [50]. These pathways have been extensively reported to regulate inflammatory responses in MASLD.

Crucially, we demonstrated that the profibrotic activity of CD24 ^+^LCN2 ^+^ LPCs is mediated through robust interactions with the TPPP3 ^+^ COL10A1 ^+^ macrophage subset. CellChat and NicheNet analyses revealed TPPP3 ^+^COL10A1 ^+^ macrophages as the primary recipients of LPC-derived signals, including CCL2, IL-34, and CSF1. These interactions polarize macrophages toward a pro-fibrotic phenotype marked by elevated expression of TPPP3 (a microtubule dynamics regulator) and COL10A1 (a collagen isoform implicated in extracellular matrix remodeling) [ 51– 53]. Immunohistochemistry confirmed increased TPPP3/COL10A1 protein levels in fibrotic septa, with spatial co-localization observed in advanced fibrotic lesions. Importantly, our findings uniquely position LPCs as upstream regulators of macrophage activation in NASH, refining the cellular hierarchy involved in the assembly of the fibrotic niche. These results highlight the pivotal role of LPC-macrophage interactions in driving MASLD-related fibrogenesis and suggest that targeting these signaling axes may offer novel therapeutic strategies for mitigating NASH progression.

Our study extends prior observations regarding TPPP3 ^+^COL10A1 ^+^ macrophages, positioning them as therapeutic targets for stage-specific intervention in NASH fibrosis. While this study provides valuable mechanistic insights into LPC-macrophage crosstalk and highlights their reciprocal signaling in NASH, several critical questions remain unresolved. First, the temporal and causal hierarchy of LPC and macrophage activation during NASH progression remains unclear, necessitating lineage-tracing models to delineate their spatiotemporal interplay. Second, direct evidence of their physical interactions and precise molecular triggers requires further validation through spatial transcriptomics and single-cell resolution co-localization assays. Third, the translational potential of targeting LPC-macrophage signaling remains to be systematically explored in preclinical models, particularly in the context of antifibrotic therapy development. Furthermore, despite single-cell RNA-seq revealing macrophage heterogeneity in NASH, the differential crosstalk between specific LPC subpopulations and macrophage subsets (TPPP3 ^+^COL10A1 ^+^ vs conventional subsets) warrants spatial validation to precisely map niche-specific interactions. Additionally, the role of metabolic stressors—such as palmitate-induced mitochondrial ROS or ER stress—in amplifying LPC-macrophage NF-κB co-activation requires further investigation, particularly regarding organelle-specific regulatory nodes. Crucially, how these processes mechanistically regulate upstream drivers ( *e* . *g*., hepatocyte lipotoxicity) and downstream effectors ( *e* . *g*., hepatic stellate cell activation) in NASH-related fibrosis remains unresolved. Addressing these knowledge gaps will not only deepen our understanding of NASH pathogenesis but also accelerate the development of targeted therapies that disrupt pathogenic LPC-macrophage circuits while preserving tissue repair functions.

In summary, our study demonstrated that resident CD24 ^+^LCN2 ^+^ LPCs play a critical role in NASH fibrosis by activating proinflammatory macrophages expressing the fibrosis-related genes *TPPP3* and *COL10A1*. These findings increase our understanding of the cellular and molecular mechanisms underlying NASH progression and highlight potential therapeutic targets for combating fibrosis. Future research should focus on developing strategies to disrupt CD24 ^+^LCN2 ^+^ LPCs- TPPP3 ^+^COL10A1 ^+^ macrophage interactions and evaluate their efficacy in preclinical and clinical settings.

## Supporting information

24954Supplementary_Materials_0407

## Supplementary Data

Supplementary data is available at *Acta Biochimica et Biophysica Sinica* online.
