# Supplementary material for: Resident CD24 +LCN2 + LPCs aggravate fibrosis and inflammatory progression via the recruitment of TPPP3 +COL10A1 + macrophages in NASH : CD24 +LCN2 + LPCs aggravate fibrosis and inflammatory progression
Source: Acta Biochim Biophys Sin (Shanghai). 2025 May 16;57(12):2034–47. doi: 10.3724/abbs.2025081 (PMC12747972; doi:10.3724/abbs.2025081)
Supplement: 24954Supplementary_Materials_0407 [file 24954Supplementary_Materials_0407.docx]

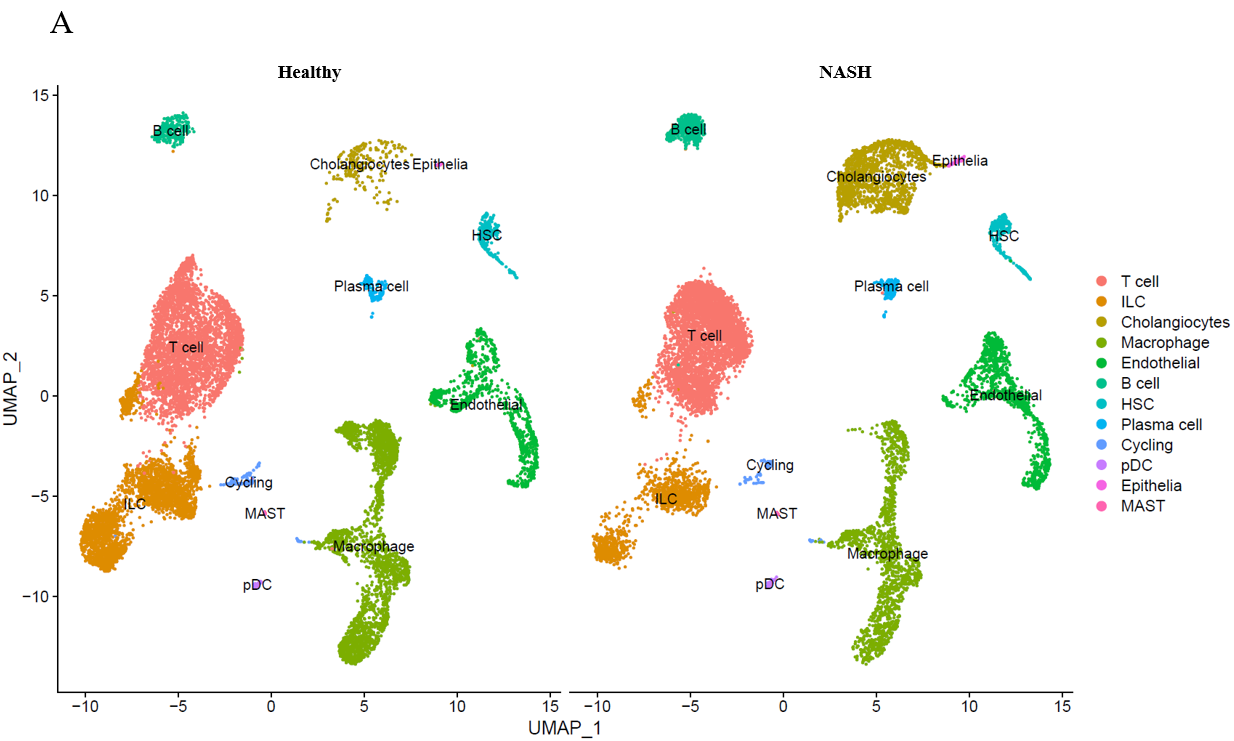


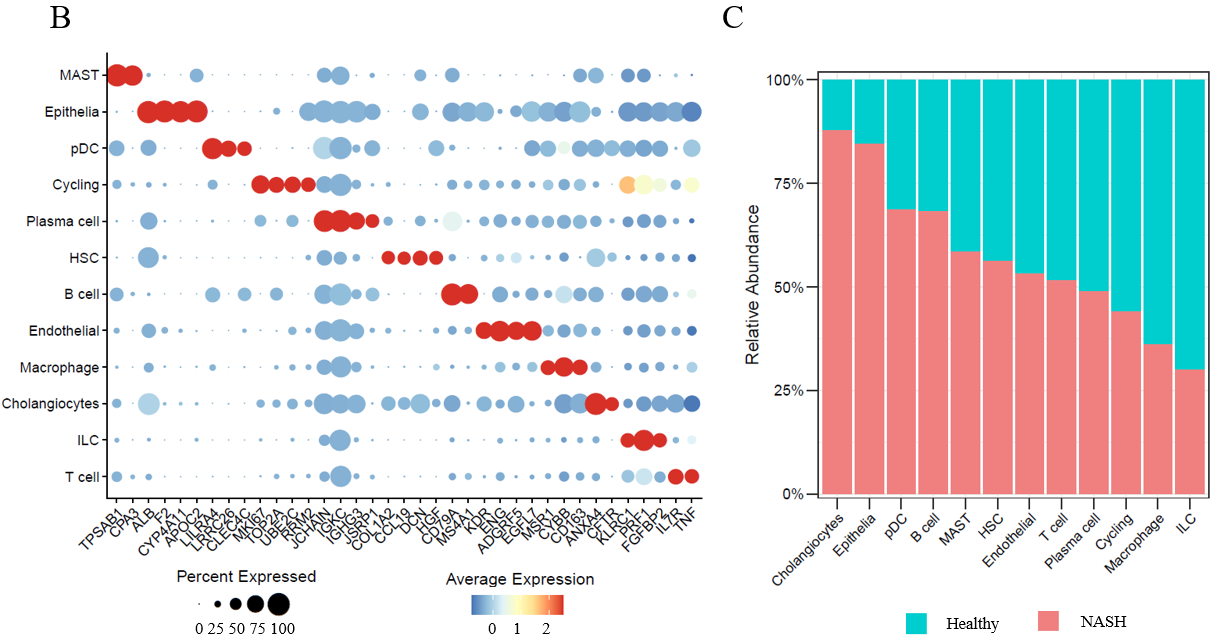


**Supplementary Figure S1. Clustering of nonparenchymal cells in Healthy and NASH livers**  (A) UMAP visualization showing the clustering of nonparenchymal cells in Healthy and NASH livers. (B) Dot plot showing the expressions of key genes in different cell clusters of livers. (C) The ratio of different cell clusters between NASH and healthy livers.


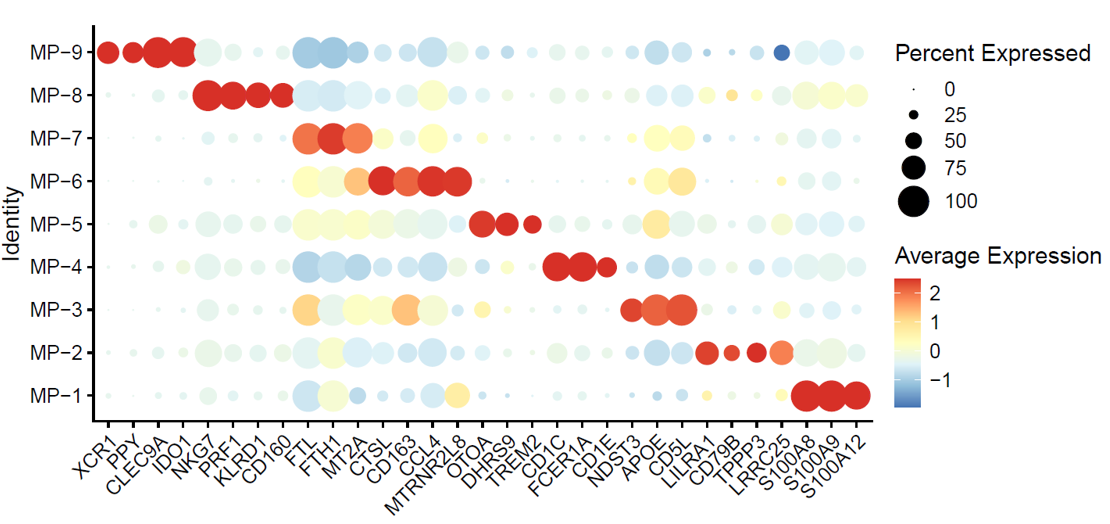


**Supplementary Figure S2. Dot plot showing the expressions of key markers for macrophage clustering in Figure 2B.**


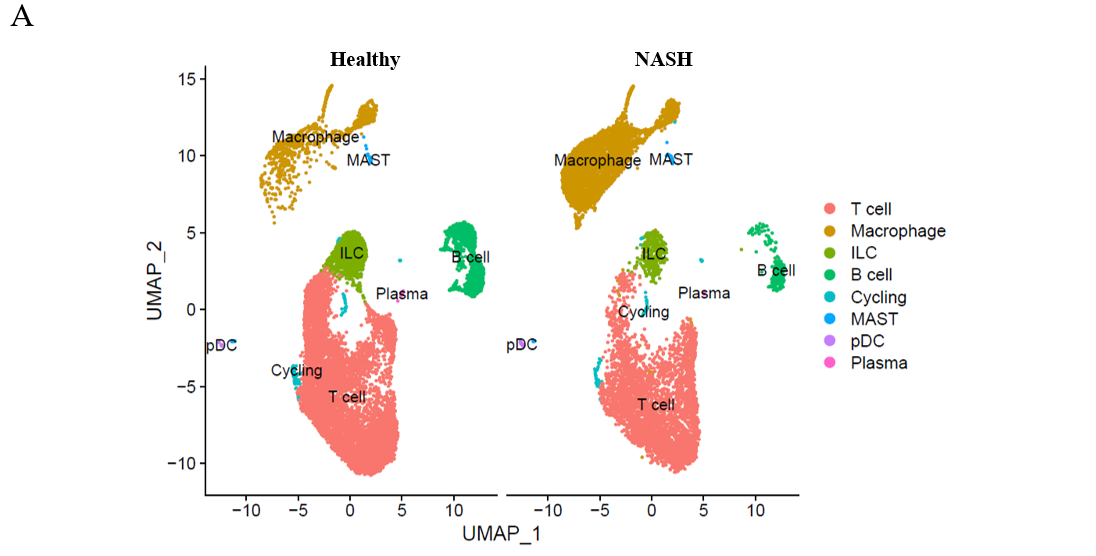


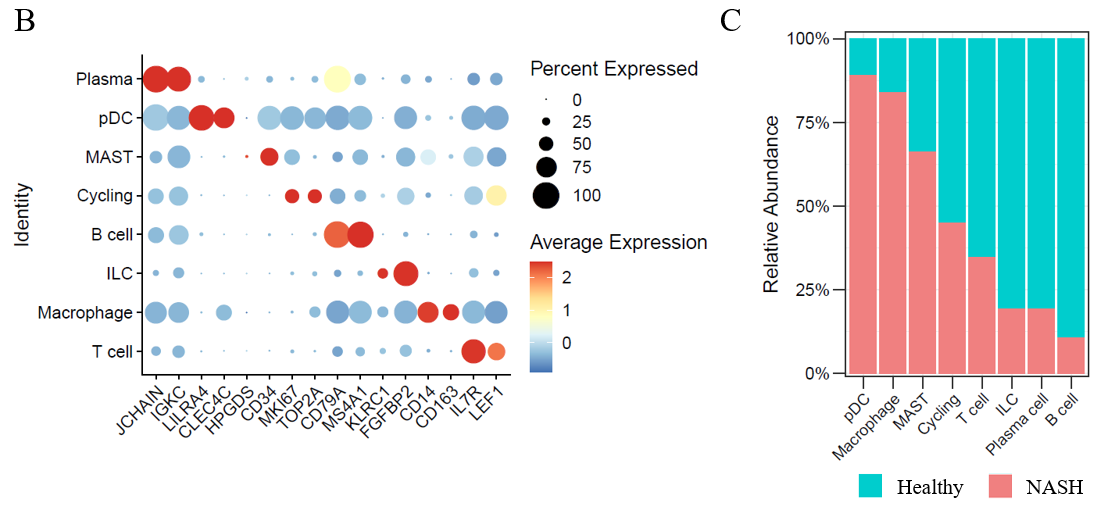


**Supplementary Figure S3**. **Recruitment of macrophages by PBMCs in** **MASLD** (A) UMAP visualization showing the integration and alignment of PBMC cell clusters from Healthy (GSM7818497-GSM7818498, 22923 cells) and MASLD (GSM4041170, GSM4041172, GSM4041173, 24111 cells) samples. The plots demonstrate good concordance between conditions. (B) Molecular markers identifying the distinct cell clusters. (C) Proportion of each cell subpopulation in Healthy and MASLD PBMCs.


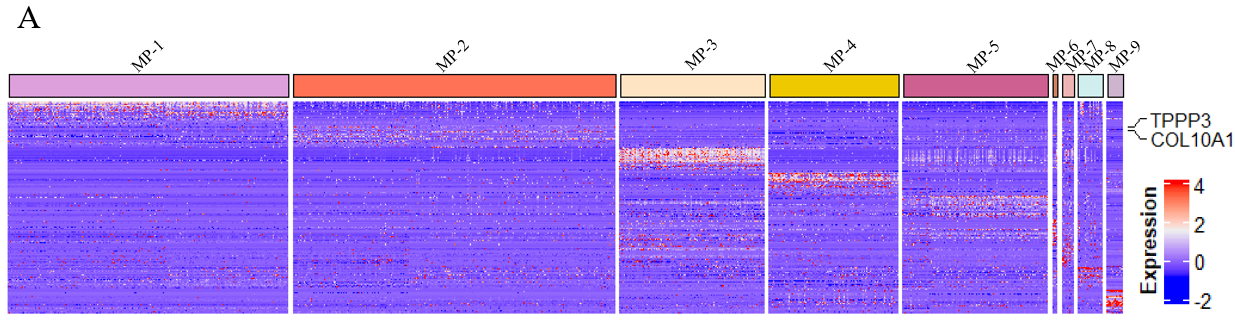


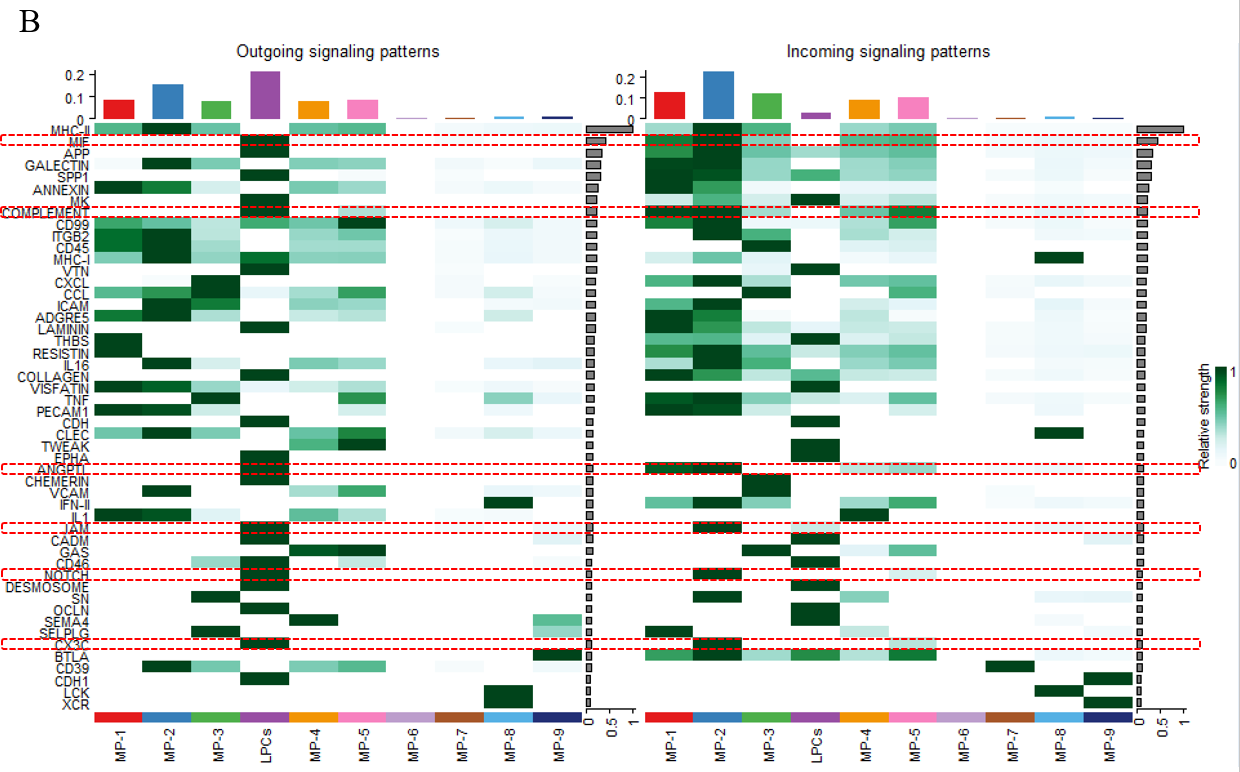


**Supplementary Figure S4.** **CellChat analysis reveals that LPCs and MP-2 macrophages primarily communicate through inflammatory signaling pathways**  (A) Heatmap of top 30 genes in MP subpopulations in MASLD. Notably, fibrosis-related genes such as *TPPP3* and *COL10A1* are highly expressed in the MP-2 subpopulation. (B) Cell-Cell communication between LPCs and other cell subpopulations in MASLD. The plot illustrates cell-cell communication between LPCs and various cell subpopulations. The red box highlights the inflammatory signaling pathways mediating communication between LPCs and MP-2 macrophages.


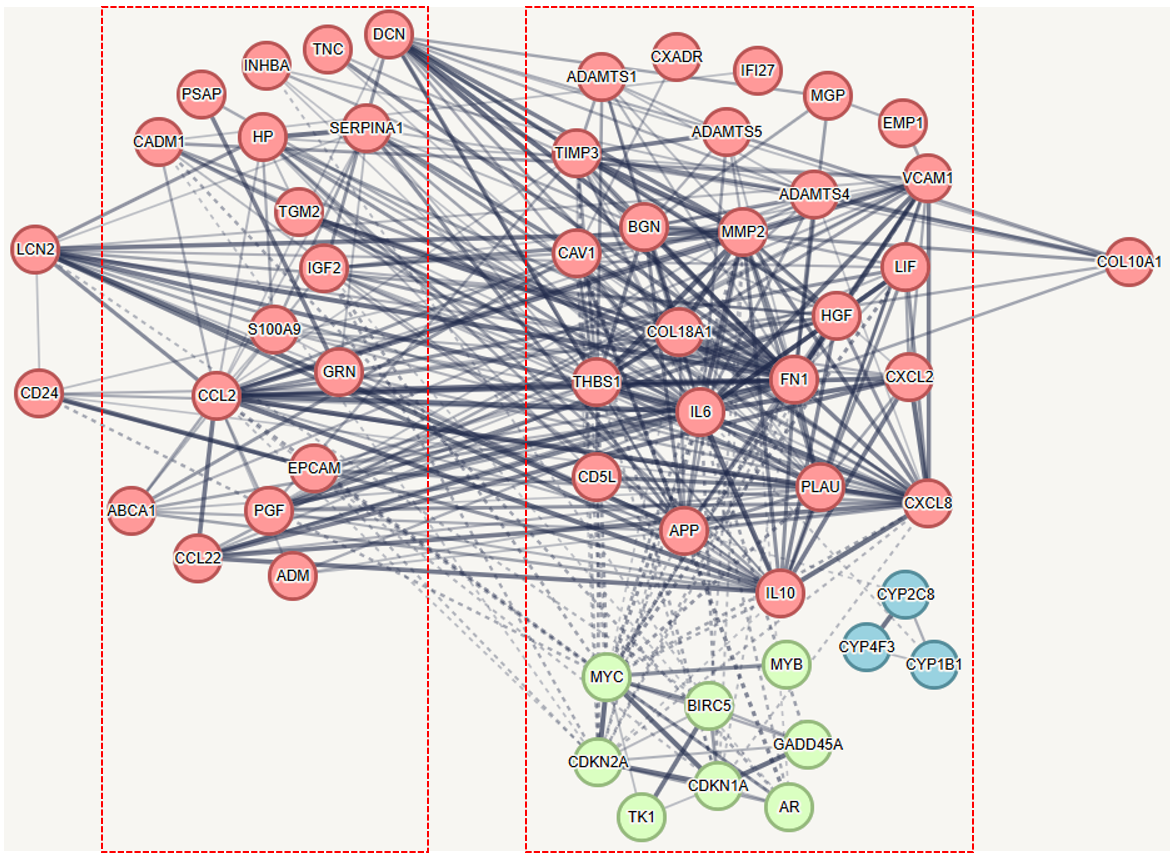

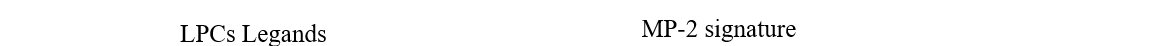


**Supplementary Figure S5. The PPI network was generated using STRING database based on the key genes identified in our single-cell RNA sequencing analysis** Nodes represent proteins, and edges represent predicted protein-protein interactions. The thickness of the edges indicates the confidence score of the interaction, with thicker edges representing higher confidence. Proteins are color-coded based on their functional clusters (red, clustered in functional pathways associated with Interleukin-4 and Interleukin-13 signaling pathway and extracellular matrix organization; green, clustered in functional pathway of chronic myeloid leukemia; blue, clustered in functional pathways associated with omega-hydroxylase P450 pathway, aromatase activity and arachidonic acid metabolism.)


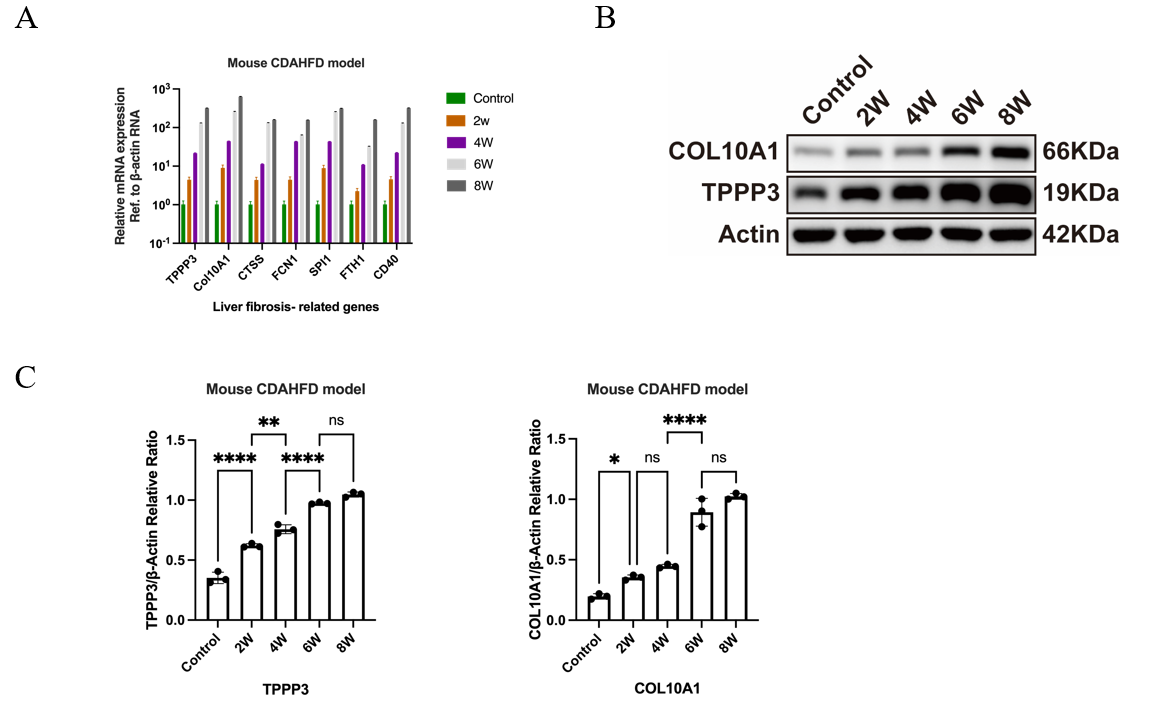


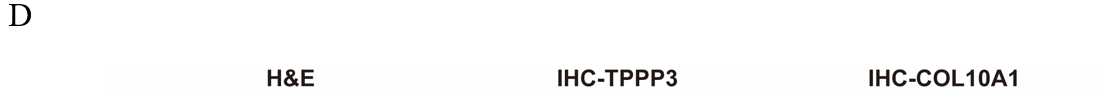


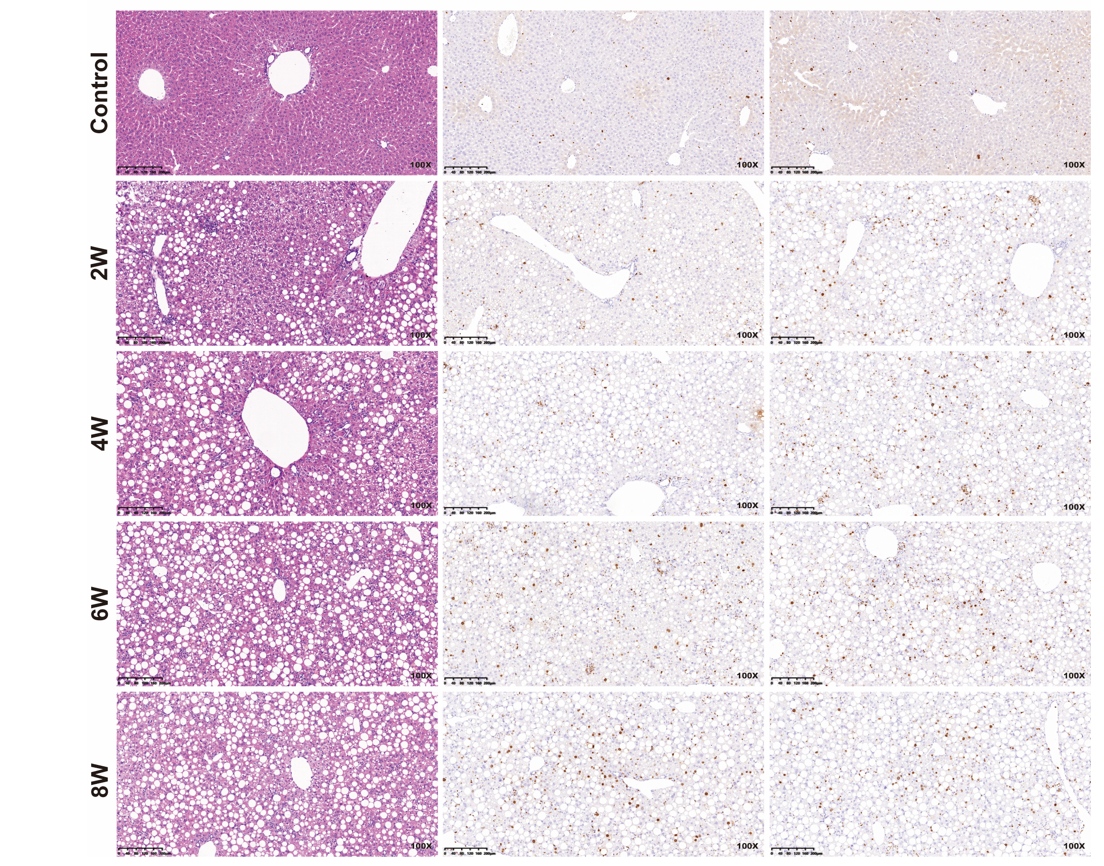


**Supplementary Figure S6.** **HFD-induced NASH mice exhibited significantly elevated TPPP3 and COL10A1 expression in areas of advanced fibrosis in their liver tissue** (A) qPCR results showing the expressions of fibrosis-related genes in HFD-induced NASH mice fed with HFD-alone for varying durations (in weeks). (B) Western blot analysis results for TPPP3 and COL10A1 expressions in HFD-induced NASH mice fed with HFD-alone for varying durations (in weeks). (C) Summarized western blot analysis data showing the relative protein levels of TPPP3 and COL10A1 in HFD-induced NASH mice fed with HFD for varying durations (in weeks). “ns” indicates no statistical significance; * *P* < 0.05, ***P* < 0.01, ****P* < 0.001, and *****P* < 0.0001. (D) Immunohistochemistry (IHC) images showing TPPP3 and COL10A1 expressions in HFD-induced NASH mice fed with HFD-alone for varying durations (in weeks). Scale bar: 200 μm.

**Supplementary Table S1. Age and gender of the four patients with clinically diagnosed MASLD and four healthy individuals**

| Patients number | Age | Gender | Healthy individuals number | Age | Gender |
| --- | --- | --- | --- | --- | --- |
| 1 | 26 | Female | **1** | 39 | Male |
| 2 | 28 | Male | **2** | 35 | Female |
| 3 | 45 | Female | **3** | 54 | Female |
| 4 | 68 | Female | **4** | 58 | Female |

**Supplementary Table S2. Antibody information**

| Antibody | Cat No. | Supplier | Purpose |
| --- | --- | --- | --- |
| TPPP3 | A22991 | Abclonal, Wuhan, China | WB |
| COL10A1 | A23884 | Abclonal | WB |
| TPPP3 | 15057-1-AP | Proteintech, Chicago, USA | WB, IHC |
| COL10A1 | #72026 | Cell Signaling Technology, Boston, USA | WB, IHC |
| β-actin | #4970 | Cell Signaling Technology | WB, IHC |
| Goat anti-rabbit-HRP | A21020 | Abbkine, Wuhan, China | WB |
| Goat anti-rabbit-HRP | G1215-200T | Servicebio, Wuhan, China | IHC |

**Supplementary Table S3. Primer sequences of human genes used in this study**

| Gene name | Forward primer (5'→3') | Reverse primer (5'→3') | Reference/source |
| --- | --- | --- | --- |
| *β -actin* | GGACTTCGAGCAAGAGATGG | AGCACTGTGTTGGCGTACAG | Designed by referring to NCBI Primer - BLAST |
| *TPPP3* | GCTGGAGATGGTGGTGAAGA | CTCTGCTGCTGGTTGTAGGT | Designed by referring to NCBI Primer - BLAST |
| *COL10A1* | CTGGTGACCTGGATGATGAG | GGTAGGTGGTGGTGTTGTAG | Designed by referring to NCBI Primer - BLAST |
| *CTSS* | CAGTGGCTGTGCTTCTGGAT | GTGCTTCCAGGGTTGTCAGT | Designed by referring to NCBI Primer - BLAST |
| *FCN1* | GACCTGCTGCTGCTGTTCTT | CGGTTGCTGCTGGTGTTGTT | Designed by referring to NCBI Primer - BLAST |
| *SPI1* | CCCAGCTGCTGCTGCTGTTCT | GGAGGGTTGCTGCTGGTGTT | Designed by referring to NCBI Primer - BLAST |
| *FTH1* | GCAGCTGCTGCTGCTGTTCT | CGGTTGCTGCTGGTGTTGTT | Designed by referring to NCBI Primer - BLAST |
| *CD40* | CAGCAGCTGCTGCTGTTCTT | GGGTTGCTGCTGGTGTTGTT | Designed by referring to NCBI Primer - BLAST |

The primer sequences in the above tables are for illustrative purposes. For actual experiments, please design and validate primers according to specific gene sequences and experimental requirements.

**Supplementary Table S4. Primer sequences of mouse genes used in this study**

| Gene name | Forward primer (5'→3') | Reverse primer (5'→3') | Reference/source |
| --- | --- | --- | --- |
| *β -actin* | TGGAATCCTGTGGCATCCATGAAAC | TAAAACGCAGCTCAGTAACAGTCCG | Designed by referring to NCBI Primer - BLAST |
| *TPPP3* | GGAGCTGCTGGTGAAGAAGGA | CTGGGTGTAGGTGAGGTTGG | Designed by referring to NCBI Primer - BLAST |
| *COL10A1* | GGACCCAGATGAAGCTGACA | GTGGTGGTTCTGGTCATGGT | Designed by referring to NCBI Primer - BLAST |
| *CTSS* | CTGGCTGTGCTTCTGGATGT | GCTTCCAGGGTTGTCAGTGT | Designed by referring to NCBI Primer - BLAST |
| *FCN1* | AACCTGCTGCTGCTGTTCTT | GGTTGCTGCTGGTGTTGTTT | Designed by referring to NCBI Primer - BLAST |
| *SPI1* | CAGCTGCTGCTGCTGTTCTT | GGTTGCTGCTGGTGTTGTTT | Designed by referring to NCBI Primer - BLAST |
| *FTH1* | CAGCTGCTGCTGCTGTTCTT | GGTTGCTGCTGGTGTTGTTT | Designed by referring to NCBI Primer - BLAST |
| *CD40* | CAGCTGCTGCTGCTGTTCTT | GGTTGCTGCTGGTGTTGTTT | Designed by referring to NCBI Primer - BLAST |

The primer sequences in the above tables are for illustrative purposes. For actual experiments, please design and validate primers according to specific gene sequences and experimental requirements.
